# Baseline frailty status and outcomes important for shared decision-making in older adults receiving transcatheter aortic valve implantation, a prospective observational study

**DOI:** 10.1007/s40520-020-01525-z

**Published:** 2020-03-19

**Authors:** Elisabeth Skaar, Anja Øksnes, Leslie Sofia Pareja Eide, Tone Merete Norekvål, Anette Hylen Ranhoff, Jan Erik Nordrehaug, Daniel Edward Forman, Andreas W. Schoenenberger, Karl Ove Hufthammer, Karel Kier-Jan Kuiper, Øyvind Bleie, Erik Jerome Stene Packer, Jørund Langørgen, Rune Haaverstad, Margrethe Aase Schaufel

**Affiliations:** 1 Department of Heart Disease, Haukeland University Hospital, Postboks 1400, 5021 Bergen, Norway; 2 Department of Clinical Science, University of Bergen, Bergen, Norway; 3 Institute of Health and Social Science, Western Norway University of Applied Sciences, Bergen, Norway; 4 Section of Geriatric Cardiology, University of Pittsburgh, Medical Center, and Geriatric Research, Education, and Clinical Center, VA Pittsburgh Healthcare System, Pittsburgh, USA; 5 Department of Geriatrics, Inselspital, Bern University Hospital, and University of Bern, Bern, Switzerland; 6 Centre for Clinical Research, Haukeland University Hospital, Bergen, Norway; 7 Department of Thoracic Medicine, Haukeland University Hospital, Bergen, Norway

**Keywords:** Aortic stenosis, TAVI, Activity of daily living, Shared decision making, Older adults

## Abstract

**Aims:**

The objective of this study was to examine baseline frailty status (including cognitive deficits) and important clinical outcomes, to inform shared decision-making in older adults receiving transcatheter aortic valve implantation (TAVI).

**Methods and results:**

We conducted a prospective, observational study of 82 TAVI patients, recruited 2013 to 2015, with 2-year follow-up. Mean age was 83 years (standard deviation (SD) 4.7). Eighteen percent of the patients were frail, as assessed with an 8-item frailty scale. Fifteen patients (18%) had a Mini-Mental Status Examination (MMSE) score below 24 points at baseline, indicating cognitive impairment or dementia and five patients had an MMSE below 20 points. Mean New York Heart Association (NYHA) class at baseline and 6 months was 2.5 (SD 0.6) and 1.4 (SD 0.6), (*p* < 0.001). There was no change in mean Nottingham Extended Activities of Daily Living (NEADL) scale between baseline and 6 months, 54.2 (SD 11.5) and 54.5 (SD 10.3) points, respectively, mean difference 0.3 (*p* = 0.7). At 2 years, six patients (7%) had died, four (5%,* n* = 79) lived in a nursing home, four (5%) suffered from disabling stroke, and six (7%) contracted infective endocarditis.

**Conclusions:**

TAVI patients had improvement in symptoms and maintenance of activity of daily living at 6 months. They had low mortality and most patients lived in their own home 2 years after TAVI. Complications like death, stroke, and endocarditis occurred. Some patients had cognitive impairment before the procedure which might influence decision-making. Our findings may be used to develop pre-TAVI decision aids.

## Introduction

Transcatheter aortic valve implantation (TAVI) is an established treatment for severe and symptomatic aortic stenosis in patients not eligible for open heart surgery, and it improves symptoms and increase life expectancy [1, 2]. While indication for TAVI has expanded to also include younger and lower risk groups, the majority of TAVI patients are older and have significant comorbidity and frailty that contraindicated surgery. The Valve Academic Research Consortium-2 (VARC 2) consensus document recommends evaluation of independence in activity of daily living before the procedure as risk stratification [3]. However, few studies have focused on independence in activity of daily living as an outcome measure [4, 5].

Among older adults who are seriously ill, death might not be feared as the worse outcome. For many, reduced quality of life and functional or cognitive impairment [6, 7], are relatively greater concerns. Dementia is highly prevalent in older adults [8] and is the leading cause of dependency in older age [9]. Shared decision-making is the favoured model for health care decisions, enhancing treatment choices to reflect patients’ values and preferences [10]. However, the context of cognitive impairment or dementia makes it more difficult for TAVI candidates to participate in the decision-making process [11].

Thus, the aim of this study was to examine baseline frailty status including cognition and outcomes important to decision-making prior to TAVI.

## Methods

### Study design

This is a single-centre prospective, observational cohort study of 82 elective TAVI patients, with 2-year follow-up. Ninety-four patients were eligible for inclusion, five refused to participate and seven were not included due to logistical reasons. The patients were recruited consecutively from 2013 to 2015.

### Participants

Patients ≥ 70 years with symptomatic and severe aortic stenosis accepted for TAVI at Haukeland University Hospital in Western Norway were included. The hospital has a tertiary function for TAVI and serves a population of 1.1 million inhabitants. Severe aortic stenosis was defined by echocardiography according to the European Society of cardiology (ESC) guidelines [1]. Before accepting patients for TAVI, a heart team including interventional cardiologists, cardiac surgeons and imaging specialists, had declined them for surgical aortic valve replacement due to high risk. At the time of the study, there was no screening for frailty preceding the intervention, and the frailty score of the study patients was not known to the treatment team. The number of patients selected for conservative treatment is not known but suspected to be small. Nursing home patients and patients with severe dementia are rarely referred for valve intervention in Norway. Exclusion criteria were inability to understand or speak Norwegian.

### Procedural characteristics

TAVI was delivered by different routes, seventy five (92%) trans-femoral, five trans-subclavian and two with direct aortic access. Two different valves were used, the Medtronic Core Valve in 52 (63%) and Boston Scientific LOTUS Valve in 30 (37%) patients.

## Measurements

### Baseline

Mini-Mental Status Examination (MMSE) assesses cognition as a scale that ranges from 0 to 30, with higher scores indicating better cognition [12]. The cut-off for normal cognitive function is usually set at 24, with a sensitivity of 0.85 and a specificity of 0.90 for identifying anyone with dementia [13].

The research team developed an 8-item frailty scale [14]. The total score is calculated by adding different domain scores: cognition (MMSE ≥ 27 = 0 points, MMSE 20–26 = 1 point, MMSE ≤ 19 = 2 points), instrumental activity of daily living (NEADL ≤ 43 = 1 point), nutrition (BMI < 20.5 = 1 point), modified SOF (low energy = 1 point, weight loss (reported, not measured weight loss; therefore, modified) = 1 point, chair stand, not able = 1 point) [15], Charlson Comorbidity Index (≥ 3 = 1 point) [16], and psychological factors (HADS ≥ 15 = 1 point) [17]. The score range from 0 to 9, were 9 represents the frailest patients. Patients were classified frail if they scored ≥ 4 points. This cut-off had a specificity of 80% and sensitivity of 60% at predicting 2-year mortality for this population. In a receiver operating curve, the area under the curve was 0.81 (95% confidence interval 0.71–0.90) [14].

### Baseline and 6 months:

*Nottingham Extended Activity of Daily living scale (NEADL)* is a scale originally developed to assess activities of daily living (ADL) for stroke patients discharged to home, yet frequently used in non-stroke populations [18, 19]. The scale measures extended activities of daily living using a 22-item questionnaire, evaluating four different sections: mobility, kitchen, domestic and leisure. Patients are asked whether they perform the different activities, and the response categories are 0 = not at all, 1 = with help, 2 = on my own with difficulty, 3 = on my own. The different scores sums into a total score of 0 to 66 points, with higher scores indicating greater independence. A score ≤ 43 indicates dependence [19]. One study of stroke patients reported a valid and reliable change if the NEADL improved or deteriorated by 4.9 points or more, and clinically important if the mean change score was in the range from 2.4 to 6.1 points after treatment [20].

*The New York Heart Association (NYHA)* classification describes patients’ symptoms of heart failure [21] and has been applied to TAVI patients [3]. NYHA class I represents no symptoms of heart failure and no limitation of physical activity, while NYHA class IV represents symptoms of heart failure at rest. Despite limitations it’s widely used both in research and clinical practice.

### Follow-up measurements

Baseline NEADL and NYHA were assessed and then repeated at 6 months. Due to some patients living far from the hospital, we performed telephone interviews at 6 months, and MMSE and frailty testing were, therefore, not conducted. For the first 2 years, we recorded composite endpoints as recommended by the Valve Academic Research Consortium-2 (VARC-2) consensus document. We collected data of admission to long-term nursing homes for the first 2 years.

### Statistical analyses

We present the data as means and standard deviations (SD), counts and percentages. Changes from baseline to 6 months are analysed using paired *t*-tests. There were little missing data so we have used complete case analysis and report the number of observations each analysis is based on. Statistical analysis was carried out in IBM SPSS Statistic 24 and R version 3.6.0 [22]*. p* values < 0.05 were considered statistically significant.

## Results

### Baseline data

#### General characteristics

We examined 82 patients with severe and symptomatic aortic stenosis, 39 (48%) women. Mean age was 83 years (SD 4.7), two patients were over 90 years, the oldest 95 years old, and there were six patients under 75 years. Most patients (62%) lived with their spouse. A majority (55%) had only primary school, while 20% had a university degree.

#### Geriatric characteristics

Fifteen patients (18%) had an MMSE score < 24, suggesting they were cognitively impaired. One fifth of the patients had a low NEADL score (≤ 43), implying dependence in at least one instrumental activity of daily living. As expected, NEADL and MMSE was correlated (Spearman’s rho = 0.47, *p* < 0.001). Eleven patients had a low BMI, however, 27 (33%) reported weight loss last year. Charlson comorbidity index was ≥ 3 in 36 (44%) of the patients, demonstrating a high burden of comorbidity. Six (7%) patients had a score ≥ 15 on the Hospital Anxiety and Depression Scale; i.e., few patients had severe anxiety or depression. For 80 patients, baseline 8-item frailty scale was calculated, and 14 patients (18%) were defined as frail.

#### Cardiovascular characteristics

Logistic EuroSCORE [23] was below 10 (predicting low surgical risk) in 20 (24%) and over 20 (high surgical risk) in 19 (23%) of the patients. Almost half (48%) of the patients had NYHA ≥ 3 at baseline, indicating a significant burden of symptoms. Few patients (11%) had a pacemaker before TAVI, and 26 (32%) had atrial fibrillation at baseline (Table 1).

**Table 1:** Patient baseline characteristics (*n* = 82)

	Mean or count	SD or (percent)
Characteristics		
Age, years	83	4.7
Women	39	(48)
Living alone	29	(35)
Education		
Primary School	45	(55)
High School	21	(26)
University	16	(20)
Geriatric characteristics		
Cognition		
MMSE	26.2	3.4
MMSE ≥24	67	(82)
MMSE 20–23	10	(12)
MMSE <20	5	(6)
Activities of daily living		
NEADL ≤43	15/80	(19)
Nutrition		
BMI	24.8	3.6
BMI <20.5	11	(13)
mSOF		
Weight loss^a^	27	(33)
Low energy	32	(39)
Unable to chair stand	10	(12)
Comorbidity		
Charlson Comorbidity Index	2.6	1.4
Charlson Comorbidity Index ≥3	36	(44)
Psychological factors		
HADS ≥15	6/81	(7)
Frailty^b^	14/80	(18)
Cardiovascular characteristics		
Logistic EuroSCORE	15	7.9
Aortic Valve Area Index, cm^2^/m^2^	0.4	0.12
Mean Aortic Valve gradient, mmHg	50	15
Left ventricular ejection fraction	57	12
NYHA ≥III	39	(48)
Previous myocardial infarction	15	(18)
CABG	15	(18)
Permanent pacemaker	9	(11)
Atrial fibrillation	26	(32)
Pulmonary hypertension	27	(33)
Cerebral Vascular Disease	12	(15)
Comorbidity		
COPD	19	(23)
Kidney failure; creatinine >177 µmol/L^c^	4	(5)

The total score is calculated by adding the different domain scores

*MMSE* mini-mental status examination, *NEADL* Nottingham extended activities of daily living scale, *BMI* body mass index, *SOF* study of osteoporotic fractures, *HADS* hospital anxiety and depression scale

^a^Modified from the original SOF, with patient-reported weight loss past year, not measured as in the original index (*mSOF* modified SOF)

^b^8-item geriatric assessment frailty scale. Missing information to calculate the scale in two patients, NYHA, New York Heart Association functional classification of heart failure, range from I to IV, most severe symptoms at IV. *CABG* coronary artery bypass grafting, *COPD* chronic obstructive pulmonary disease

^c^As reported in the PARTNER study [2]; Creatinine > 2 mg/dl (177 µmol/L)

### Follow-up

One patients was lost to follow-up at the telephone interview and three had died. For three patients, information was missing on whether they lived at home or in nursing home at 2-year follow-up. VARC 2 composite endpoint is presented in Table 2, and endocarditis is separately presented in Table 3 with complementary data.

**Table 2:** Composite endpoints according to VARC 2^a^ criteria

	Total (*n* = 82)	Percent
*Device success*		
Absence of immediate procedural mortality^b^	82	100
Correct positioning	81	99
Intended performance of the prosthetic heart valve^c^	81	99
No moderate or severe prosthetic valve regurgitation^d^	78	95
*Early safety (at 30 days)*		
All-cause mortality	2	2
All stroke(disabling or non-disabling) in hospital^e^	1	1
Life-threatening or disabling bleeding	4	5
Acute kidney injury-stage 2 or 3^f^	2	2
Coronary artery obstruction requiring intervention	1	1
Major vascular complication	5	6
Valve-related dysfunction requiring intervention	1	1
*Clinical efficacy (30 days–2 years)*		
All-cause mortality	4	5
All stroke(disabling or non-disabling)	5	6
Requiring hospitalizations for valve-related symptoms or worsening congestive heart failure	13	16
NYHA class III or IV^g^	4	5
*Time-related valve safety*		
Structural valve deterioration		
Valve-related dysfunction (mean aortic valve gradient ≥20 mmHg) and/ or moderate or severe prosthetic valve regurgitation^h^	15/81	19
Requiring repeat procedure (TAVI or SAVR)	1	1
Prosthetic valve endocarditis^f^	6	7
Trombo-embolic events (eg stroke)	6	7
VARC bleeding (life threatening/disabling bleeding or major bleeding), unless clearly unrelated to valve therapy (e.g. trauma)	14	17

*SAVR* Surgical aortic valve replacement, *TAVI* transcatheter aortic valve implantation, *NYHA* New York Heart Association

^a^The Valve Academic Research Consortium (VARC)-2 consensus document (see references)

^b^Immediate or consequent death ≤ 72 h post-procedure

^c^No prosthesis-patient mismatch. Mean aortic valve gradient < 20 mmHg or peak velocity < 3 m/s

^d^After TAVI procedure at index hospitalization

^e^Assessment of stroke at index. All strokes verified by CT/MRI

^f^Evaluation of acute kidney injury is based on serum creatinine, we miss data on urine output

^g^NYHA at 6 months

^h^Four patients had new paravalvular leak or vegetation on the aortic valve

**Table 3:** Infective endocarditis first 2 years after TAVI

TAVI valve	Age at TAVI and gender^a^	Days after TAVI, positive blood culture	Bacteria (4/4 blood cultures)	Dukes criteria^b^	Outcome two year	TTE and TEE	Pacemaker device
Lotus 27 mm	86 y, F	49	*Stafylococcus aureus*	1 Major + 3 minor	Dead ^c^	No vegetation or PVL	PM, 1 day after TAVI
Core valve 31 mm	77 y, M	190	*Streptococcus salvarius*	2 Major + 2 minor	Alive	Aortic valve vegetation	CRT-D , 3 months before TAVI
Core valve 31 mm	80 y, M	380	*Enterococcus faecalis*	1 Major + 3 minor	Alive	New aortic PVL	CRT-P, 2 months after TAVI
Core valve 29 mm	79 y, M	407	*Streptococcus sanguinis*	1 Major + 3 minor	Alive	New aortic PVL	No
Core valve 31 mm	77 y, M	448	*Stafylococcus aureus*	2 Major + 4 minor	Dead ^d^	Aortic valve vegetation	CRT-P, 1 year before TAVI
Core valve 31 mm	80 y, M	528	*Streptococcus oralis*	1 Major + 1 minor	Alive	No vegetation or PVL	PM, 1 day after TAVI

*TTE* transthoracic echocardiography, *TEE* trans-oesophageal echocardiography, *PVL* para valvular leak, *PM* pacemaker, *CRT-P/D* coronary resynchronisation therapy with cardiac defibrillator (D) or pacemaker (P)

^a^*y* years, *M* male, *F* female

^b^All meet criteria for definite infective endocarditis (IE) except the last patient who has a possible IE. All patients had trans-femoral access for TAVI

^c^Died five months after diagnosed with IE

^d^Died one month after diagnosed with IE

### Mortality and morbidity

Two patients (2%) died early (< 30 days), and one had a disabling stroke. At 1 year, four patients (5%) had died and 4 (5%) had a disabling stroke. After 2 years, 6 (7%) patients had died, five of cardiovascular and one of non-cardiovascular cause. Four patients lived in nursing homes. There were no new disabling stroke from one to 2 years. Six patients (7%) had endocarditis during the first 2 years. Thirty-two patients (39%) got a new pacemaker perioperative (during the hospital stay).

### Patient-reported outcome measures at 6 months

#### NEADL

The NEADL score was available in 78 patients at 6 months. All patients were reached in person except for one, where the spouse provided information. For one patient, only NEADL at 6 months was available. There was no change in mean NEADL (*n* = 77) at baseline and 6 months with 54.2 (SD 11.5) and 54.5 (SD 10.3), respectively, mean difference 0.3 (*p* = 0.7). Even so, 13 patients (17%) improved 5 points or more at the NEADL from baseline to 6 months, and 14 patients (18%) deteriorated 5 points or more. We did not find an association between frailty status and deterioration in NEADL score. For example, the proportion of patients who deteriorated in NEADL score was similar in the frail and the non-frail group (42% and 55%, respectively, *p* = 0.53, Fisher’s exact test).

#### NYHA class

**Fig. 1:**
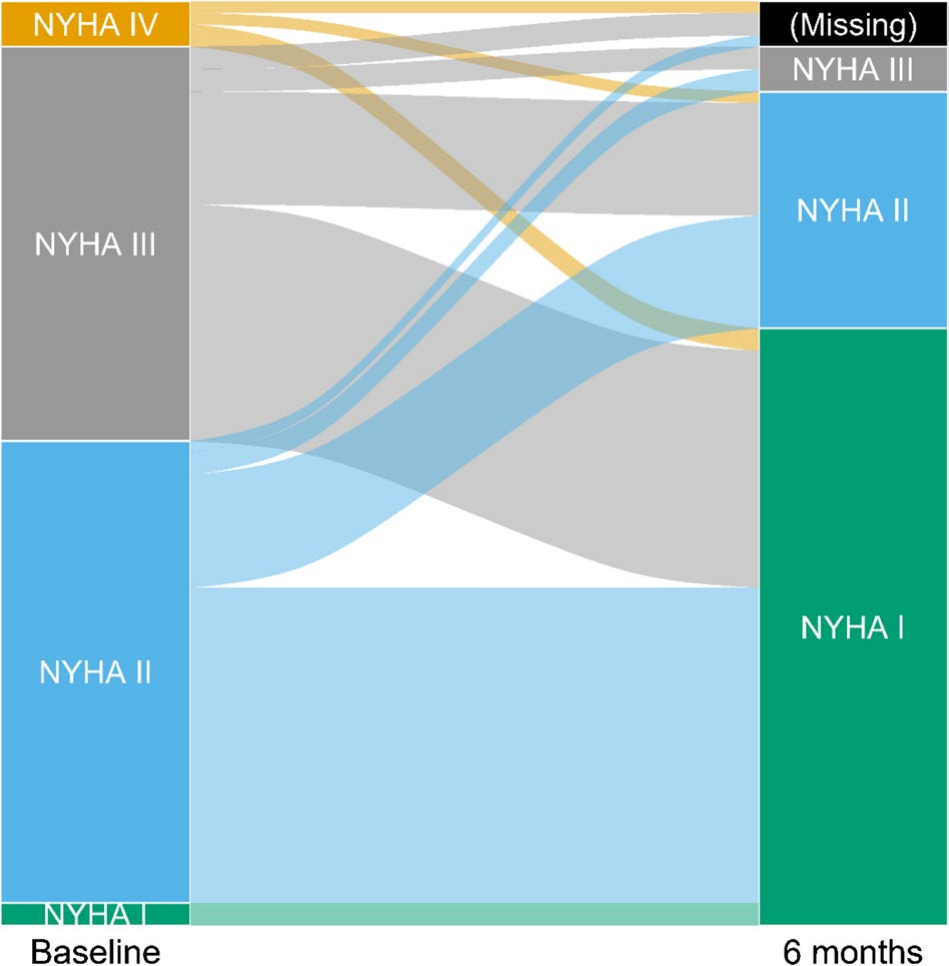
Individual changes in NYHA class from baseline (*n* = 82) to 6 months (*n* = 78). The height of each bar is proportional to the number of patients with the corresponding NYHA class, and the width of the ends of each flow line is proportional to the number of patients with the given pattern of change of NYHA class

After 6 months, the majority was in NYHA I (68%), about a quarter were in NYHA II (27%) and a few in NYHA III (5%). No patients were in NYHA IV. Mean NYHA (*n* = 78) at baseline and 6 months was 2.5 (SD 0.6) and 1.4 (SD 0.6), respectively, a significant improvement (*p* < 0.001). Four patients had missing NYHA class at 6 months (three dead, one lost to follow-up) (Fig. 1).

## Discussion

This prospective observational study of 82 patients documents symptom improvement and maintenance of activities of daily living 6 months after TAVI. They had low mortality and most patients lived in their own home 2 years after TAVI. At baseline, 18% had an MMSE score, indicating cognitive impairment or dementia. We found a higher frequency of endocarditis than expected. According to the basic principles of shared decisions-making, balanced information regarding risks and benefits, and exploring patients’ values and goals are important. Physicians should be aware that patients’ cognitive impairment or dementia might affect the ability to participate in the decision-making process and give an informed consent.

Mortality at one (5%) and 2 years (7%) after TAVI was low in this cohort, where mean age is over 80 years and there is a substantial burden of comorbidity and frailty. We found a substantial improvement in NYHA class from baseline to 6 months. Based on the improvement of symptoms we expected to find an improvement in NEADL. However, we found no change in mean NEADL. There was a variation on an individual level. Some patients improved their independence and others deteriorated, although for most patients, the change in NEADL was minor. Other factors, like frailty and dementia, probably have more impact on the level of independence than the aortic stenosis per se [4], and 6 months follow-up is probably too short to establish deterioration due to other causes. There was no difference between the frail and robust patients regarding change in NEADL from baseline to 6 months.

At baseline, 15 patients had an MMSE below 24, indicating possible cognitive impairment or dementia [13]. Five patients had an MMSE less than 20, which increases the probability of incapacity, and likely reduces their power of judgement during the decision-making processes before TAVI [11]. Several studies have suggested that a low MMSE score at baseline predicts poor outcomes after TAVI [4, 24]. However, there are also studies demonstrating cognitive improvement after TAVI when impairment is caused by the aortic stenosis itself [25]. For patients with established dementia, surviving to end stage dementia might not be what the patient would choose autonomously. These are difficult issues to discuss with patients with dementia and their families, both due to patients’ anosognosia (a physiological damage to the brain, where patients have no awareness of their disease) [26], and also health care professionals’ fear of patients losing hope when focusing on the deterioration and increased dependence expected after a diagnosis of dementia [27]. It is important to point out that an MMSE < 24 is not diagnostic of dementia, and sensitivity and specificity for MMSE cut-offs in a TAVI population, might be different than in the general population. Suspicion of dementia should, therefore, lead to further investigations.

We found a higher frequency of infective endocarditis (IE) than expected. In a recent meta-analysis with a mean follow-up at 3.4 years, the overall incidence of IE in TAVI was 2.0% [28]. In the present study, five of six patients with IE had a pacemaker or an ICD, which might be associated with an increased risk of IE [29]. Several single-centre studies finding higher incidences suggests that IE is underreported in large studies and registries [30, 31]. Diagnosing endocarditis is challenging, and the presentation might be uncharacteristic since the patients are old with comorbidities [28]. As endocarditis can occur later on, longer follow-up than 1 year seems important. Antibiotic prophylaxis during the TAVI procedure were administered to all patients. General practitioners were informed routinely on discharge about the risk of endocarditis and indications for prophylaxis.

Prior to TAVI, patients need to be informed of expected NYHA class improvement, survival benefit and maintenance of activities of daily living. Rare and severe complications, like death, stroke and infectious endocarditis, should also be part of the pre- TAVI discussion with the patient. Old patients with substantial comorbidity need to be informed that TAVI will not solve all their health problems. After the study period, written information about the procedure and risks and benefits was developed and administered to patients as part of the decision-making process. Our findings may be used when developing and improving decision aids for this treatment in older adults.

### Strengths of the study

All hospitals in Western Norway use the same electronic medical records and VARC-2 endpoints are complete with no patients lost to follow-up. All deaths are automatically registered in the patients’ electronic journal. The same investigator (ES) performed the assessment at baseline and 6 months, increasing reliability.

### Limitations of the study

This is a single centre study performed in the early era of TAVI and some of the results might not be transferable due to improvement of equipment, better patient selection and training of the interventional cardiologists performing the procedure. Most patients were independent before TAVI, and minor (nevertheless important to patients) improvements might not be revealed by the NEADL questionnaire. Due to the small sample size, it was not possible to analyse whether specific subgroups improved or deteriorated in NEADL. We performed only telephone interviews at 6 months, preventing us from assessing MMSE and frailty at follow-up. We assume that living in their own home 2 years after TAVI reflects independence of activities of daily living, however, we cannot exclude that some patients living at home were in need of extended care.

## Conclusion

TAVI patients had symptom improvement and maintenance of activity of daily living at 6 months. They had low mortality, and most patients lived in their own home 2 years after TAVI. Severe complications, like death, stroke and endocarditis, occurred. Balanced information regarding these risks and benefits is needed to ensure informed consent prior to the procedure, and our findings may be used to develop and improve decision aids assisting this process. Clinicians should be aware that some patients have cognitive impairment before TAVI that might affect their power of judgement and decision-making.
